# Multi-Center Two-Year Patency Outcomes of Endovascular Arteriovenous Fistulas (endoAVF) Created with a 4 French System

**DOI:** 10.1007/s00270-024-03754-5

**Published:** 2024-06-05

**Authors:** Erez Klein, Brandon Repko, Alejandro Alvarez, Nicholas Inston, Robert Jones, Dheeraj K. Rajan

**Affiliations:** 1 Department of Medical Imaging and Interventional Radiology, Moffitt Cancer Center, Tampa, FL USA; 2 Department of Interventional Radiology, Butler Health System, Butler, PA USA; 3 Department of Internal Medicine and Nephrology, SSM Health, St. Luis, MO USA; 4 Department of Interventional Radiology, Queen Elizabeth Hospital Birmingham, University Hospital Birmingham, Birmingham, UK; 5 Department of Vascular and Interventional Radiology, University Medical Imaging of Toronto, Toronto, ON Canada

**Keywords:** Endoavf, Hemodialysis, Wavelinq, Percutaneous, Arteriovenous fistula

## Abstract

**Purpose:**

To assess multicenter two-year patency outcomes of endovascular arteriovenous fistulas (endoAVF) created with the WavelinQ device.

**Materials and Methods:**

Patients who had fistulas created at three centers from January 2018 to December 2020 were included in this retrospective study. In total, 112 patients underwent endoAVF creation [40 females, 72 males; mean age 60 years (range 18–88)]. Data collected included patient demographics, location of fistula creation, interventions performed, and brachial artery flows pre- and post-creation. Two-year cumulative patency, functional patency, and primary patency were assessed with Kaplan–Meier methodology. Factors affecting patency and maturation were examined using the Cox proportional hazards model.

**Results:**

Technical success defined as angiographically successful endoAVF creation was 97.3% (109/112). In 11 patients the fistula did not mature for dialysis use. For 98 patients (87%) with endoAVF maturation, 12- and 24-month cumulative patency was 94.3% and 91.7%. Functional patency (two-needle cannulation) at 12 and 24 months was 95.7% and 92.7%, respectively. Median maturation time is 95 days (IQR 51–231 days). Male gender and brachial vein coiling at the time of endoAVF creation were predictive of maturation. There were 34 censored events (four patients undergoing renal transplantation; 30 patients deceased). Number of reinterventions per patient year was 0.73 where 43 were maturation procedures and 101 were maintenance procedures. One Grade 3 complication occurred of arterial access puncture site pseudoaneurysm.

**Conclusion:**

A high two-year functional and cumulative patency following endoAVF creation with the WavelinQ device was observed in this multicenter real-world experience Level of Evidence: 3

*Level of Evidence* III

**Graphical Abstract:**

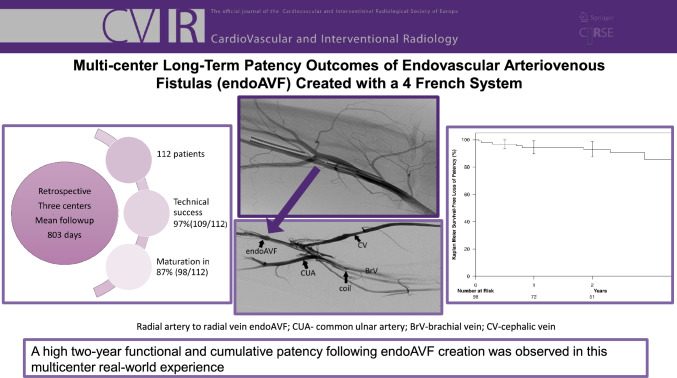

## Introduction

The most common form of kidney replacement therapy globally is dialysis (78%), and 90% of these patients are treated with hemodialysis [1]. Hemodialysis requires dedicated vascular access between the patients’ circulation and the hemodialysis machine. The recently updated Kidney Dialysis Outcomes Quality Initiative guidelines emphasize that of the two types of dedicated vascular access, an arteriovenous fistula (AVF) or arteriovenous graft is preferred over a central venous catheter [2]. However, an AVF is preferable as it is associated with fewer long-term vascular complications such as thrombosis and fewer interventions to maintain functional patency [3].

Surgical AVF creation commonly performed in an operating room requires a skin incision, creating an anastomosis with suture material between the artery and vein together. This can be performed at various levels within the upper extremity. Surgical fistulas have a primary failure rate of 20–60% and require a maturation time of approximately four months [3]. EndoAVFs do not require a surgical incision, require no anastomotic suturing, and can be performed in an angiography capable facility. Potential advantages of endoAVFs are improved outcomes, access to more operators that can create an AV access and no requirement for operating room use.

The two devices approved within North America and the European Union for endoAVF are the WavelinQ (Becton Dickinson, Franklin Lakes, NJ) and the Ellipsys (Medtronic, Minneapolis, MN) devices. Both WavelinQ and Ellipsys device rely on the presence of a venous perforator in the proximal forearm to arterialize the deep venous system and direct flow to the superficial veins. The WavelinQ device is a 4Fr dual-catheter system, with one catheter introduced arterially and the other introduced venously. Radiofrequency energy is used to create the fistula. Post-creation, brachial vein embolization is performed to enhance flow to the superficial system. The Ellipsys device is a 6-Fr catheter that is advanced through a sheath that is originally inserted via the venous perforator into the radial artery. The device uses thermal energy to create the fistula. Post-creation, the venous perforator is angioplastied to enhance flow to the superficial system. The WavelinQ system allows for creation of different fistulas in the proximal forearm but requires fluoroscopy for the procedure. The Ellipsys device allows for the creation of one type of fistula but can be created with only ultrasound guidance.

The WavelinQ device had its origins as a 6 French (Fr) device known as the TVA FLEX device. The first-in-human study and the later pivotal study with the original device proved that percutaneous creation of autogenous hemodialysis fistulas was possible with a high technical success rate, high maturation rate, and low intervention rates. However, longer-term usability and patency have not been assessed and are not relevant clinically given discontinuation of the 6 Fr device and introduction of the smaller 4 Fr BD WavelinQ device in 2019 [4, 5].

Since 2019, there have been a couple studies that have demonstrated a high technical success, maturation rate, and low intervention rates with the available 4 Fr device, but these studies are limited to short-term outcomes of 72–83% six-month primary patency [6, 7]. One study has been published with longer-term patency of 82% 24-month cumulative patency but with a limited study size of thirty patients [8]. Given the paucity of longer-term outcomes with the WavelinQ device, this study assesses combined outcomes from three centers.

## Materials and Methods

This study was approved by the institutional review board (IRB) at one center, and approval was not required at the other two due to the retrospective nature. This was a three-center retrospective investigator-sponsored study (supported by an unrestricted cybergrant from BD) of patients who underwent endoAVF creation with the 4 Fr WavelinQ EndoAVF system (Becton Dickinson, Franklin Lakes, New Jersey, USA) in the USA at one hospital-based, nonacademic practice, one outpatient center, and one UK academic center. Three operators performed the procedures, two interventional radiologists (15-year endovascular experience, 3-year endoAVF experience; 20-year endovascular experience, 6-year endoAVF experience) and one interventional nephrologist (18-year endovascular experience, 3-year endoAVF experience). Patients were referred from regional nephrologists and access surgeons. Patients were identified from institutional electronic medical record databases requiring long-term hemodialysis who underwent endoAVF creation between January 2018 and December 2020.

Referrals were assessed with ultrasound to determine whether *a* > 2 mm venous perforator connected to a deep ulnar or radial vein and a superficial cephalic and/or basilic vein. If the patients then met size requirements (> 2 mm) of the access vein and artery, as well at site of fistula creation diameters of > 2 mm, with less than 1.0 mm distance separating the artery and vein at the planned anastomotic site they underwent fistula creation (requirements within the instructions for use of the WavelinQ device are distance between target artery and vein < 1.0 mm and target vessels > 2 mm in diameter). Patients were excluded for uncorrectable coagulopathy and known central venous occlusion on the side of intended endoAVF creation.

## Procedure

Most patients had the procedure performed with moderate IV sedation (midazolam and fentanyl) with a minority undergoing brachial plexus block at one center (arbitrarily selected patients). The WavelinQ endoAVF system consists of two 4Fr catheters, one advanced into the artery and the other advanced into the vein. If a radial artery to radial vein fistula was intended, the arterial catheter and venous catheter were introduced from the wrist level. If an ulnar artery to ulnar vein fistula was intended, depending on entry vessel diameter, catheters were introduced from the ulnar artery and ulnar vein at the level of the wrist or from the brachial artery and vein in the upper arm. If the ulnar vein at the wrist level was < 2 mm, but the ulnar artery at the wrist was suitable size, the venous catheter was introduced from the brachial vein with the arterial catheter advanced retrograde from the wrist access site (anti-parallel approach). Interosseous fistulas were created by catheters introduced from the brachial artery and vein in the upper arm. A spring-loaded electrode from the venous catheter transmits a short radiofrequency (RF) pulse via a footplate traversing the two vessel walls into the artery, promoting tissue vaporization and fistula creation. Rare earth magnets and markers embedded within the catheters assist with proper alignment of the two catheters visualized fluoroscopically before delivery of the RF energy. After creation an arteriogram was performed to verify creation of the fistula (visualized as flow from the artery through the fistula created into the veins of the arm), to determine if any immediate complications occurred such as extravasation or pseudoaneurysm and to determine if brachial vein coil embolization was required to enhance flow to the superficial veins. The operator determined the need for embolization based on angiographic findings of preferential or equal venous flow to the brachial veins compared angiographically to the cephalic and basilic veins. Venous embolization if required was performed after selecting the brachial vein that was receiving dominant flow [9]. Embolization devices used to reduce flow in the brachial vein included pushable Nester and Tornado coils and Retract detachable coils (6–8 mm diameter, 7–15 cm length; Cook Medical) and the Caterpillar embolization device (4–6 mm; BD). Placement was always in the peripheral (distal) lateral brachial vein just central to the perforator vein, and confirmation of preferential venous flow post-embolization in the cephalic and/or basilic vein was assessed angiographically. Hemostasis after removal of the sheaths was obtained with digital compression (vein puncture site < 5 min; arterial puncture site < 15 min). Brachial artery flow before and after fistula creation (available for 97 patients) was obtained.

### Follow-Up Imaging and Interventions

At one center a vascular ultrasound examination was performed three weeks post-creation. At another blood flow volume in the ipsilateral brachial artery was assessed at 1-, 2-, and 6-weeks post-creation. The third center performed duplex ultrasound examination at 2- and 6-week post-endoAVF creation.

Maturation interventions were performed to facilitate usability of the endoAVF for hemodialysis. If brachial artery flow on follow-up after endoAVF creation was less than 500 ml/min and/or brachial vein flows were 200–300 cc/min or more and flows in the cephalic and/or basilic veins were less than 400-500 cc/min, a fistulogram was performed to exclude a perforator vein or juxta-anastomotic vein stenosis and treat the stenosis (at all three centers). If a large deep vein (> 4 mm) or deep collateral vein was stealing flow from the superficial veins, it was embolized to divert flow to the superficial veins. The criteria for an endoAVF to be considered ready for cannulation were a flow volume of ≥ 500 mL/min in the brachial artery and a ≥ 4-mm diameter of the vein for cannulation. When the arterialized vein segment was considered too deep for successful cannulation at the dialysis center, the patient was referred to an access surgeon and standard surgical techniques of superficialization and elevation was performed.

Maintenance interventions were those performed when the endoAVF, which had matured and was in use for hemodialysis, became dysfunctional (which included symptoms of poor dialysis clearance, decreased fistula thrill, or onset of difficulty with needle/cannulation access).

Maintenance interventions were performed from ultrasound-guided wrist artery access (radial/ulnar). A 3Fr inner cannula of a micro-puncture set was used for diagnostic angiography. If a stenosis was found (perforating vein or the juxta-anastomotic vein), arterial access was upsized to a 5 Fr sheath with moderate conscious sedation. The wrist access for intervention allows for a very straight access point for intervention allowing for easy passage of the wires and catheters through the anastomosis and into the cephalic/basilic veins. A cocktail of 2500 units of heparin, 2.5 mg verapamil, 200 μg nitroglycerin was administered through the sidearm of the sheath. If wrist arterial sheath placement was not possible due to vessel size, retrograde venous access was obtained from the cephalic or basilic vein. The stenosis was commonly traversed with diagnostic 4Fr 30 cm catheter and V18 wire (Boston Scientific, Marlborough, MA). PTA was performed with either 5–6-mm noncompliant high-pressure balloons or cutting balloons if proper effacement was not achieved with HPB.

### Statistics and Definitions

Technical success was defined as successful creation of a fistula between desired artery and vein determined angiographically with arterial injection of contrast flowing into the outflow veins of the proximal forearm. Fistula maturation was defined as the endoAVF ready for hemodialysis use based on brachial artery flow > 500 ml/min and an outflow vein diameter > 4 mm or the fistula underwent successful two-needle cannulation for at least 2/3 (67%) of dialysis sessions within a single month [10]. Primary patency was defined as time from endoAVF creation to first intervention. Cumulative or secondary patency was defined as the time from endoAVF creation to loss of the endoAVF (including surgical revision of the anastomosis). Predialysis was defined as clinical situation in which the patient has significant impairment of kidney function that will ultimately lead to either death or inclusion in kidney replacement therapy (dialysis and/or transplantation). Elevation or transposition of vein segments post-creation was considered maturation procedures and not loss of the endoAVF. Functional patency was from time of maturation with two-needle cannulation to loss of the endoAVF for continued dialysis. The Kaplan–Meier methodology was used to determine two-year cumulative and functional patency. Events censored were patients lost to follow-up, patient death, or renal transplantation. Factors affecting patency and maturation were examined using the Cox proportional hazards model. A variable was considered significant if the p value was less than 0.05.

Complications were defined according to CIRSE guidelines and graded 1–6 with 1 representing a complication during the procedure which could be solved within the same session and 6 representing death [11].

## Results

**Fig. 1:**
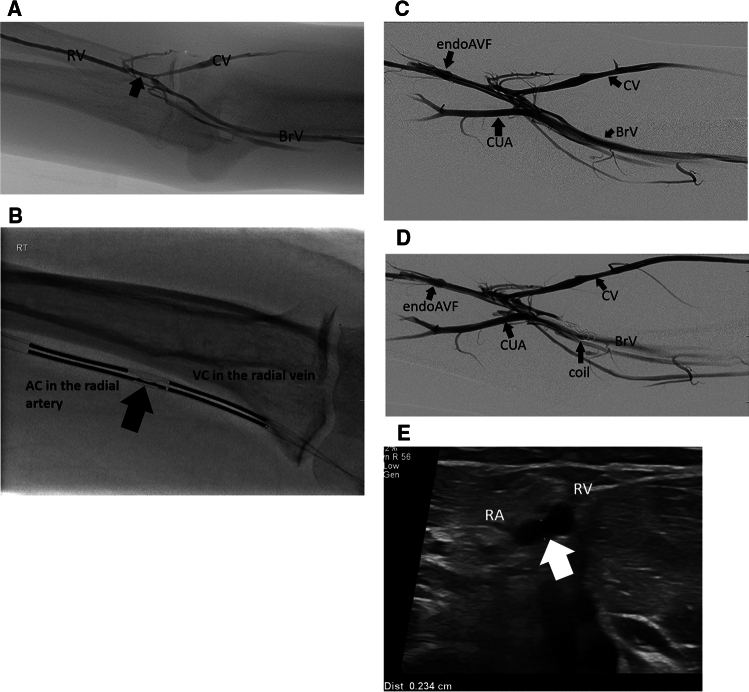
Radial artery to radial vein endoAVF. **a** Venogram of the lateral radial vein with opacification of the perforating vein (arrow) that fills the cephalic vein in the upper arm (CV) but also the brachial vein (BrV). **b** Venous catheter (VC) in lateral radial vein aligned with the arterial catheter (AC) in the radial artery (arrow showing alignment of footplate with endplate prior to endoAVF creation). **c** Post-endoAVF (arrow) creation with arteriogram performed from the radial artery demonstrating near equal venous filling of the cephalic (CV) and brachial veins (BrV) (CUA—common ulnar artery). **d** Post-coil embolization of the brachial vein (BrV). Arteriogram demonstrates preferential flow in the cephalic vein (CV) with greatly reduced flow in the brachial vein. **e** One month post-endoAVF creation, the fistula between the radial artery (RA) and radial vein (RV) is patent

One hundred and twelve patients underwent the endoAVF procedure. Technical success of the procedure was 97% (109/112) with three failures attributed to calcification of the ulnar artery at the site of intended endoAVF creation. In 11 patients out of the 109 technically successful procedures (10%), the arteriovenous fistula failed to mature for dialysis usage despite additional interventions promoting fistula maturation and patients eventually underwent surgical fistula creation with no further follow-up. Fistula maturation with subsequent use for dialysis was reported in the remaining 98 patients (demographics and characteristics are summarized in Table 1). In 65 patients (66.3%, 65/98) ulnar–ulnar arteriovenous fistula was created, in 27 patients (27.5%, 27/98) radial–radial arteriovenous fistula was created (Fig. 1), and in 6 patients (6.1%, 6/98) arteriovenous fistula was created between the interosseous artery and vein. Mean brachial artery flow before and after fistula creation (available for 97 patients) was 66 ml/min and 1099 ml/min, respectively (*p* < 0.01). In 78 patients (80.4%) coil embolization of a brachial vein was performed at time of the procedure to promote preferential blood flow through the newly created fistula.

**Table 1:** Patient demographics

	*n*	%
Male	61/98	62%
Female	37/98	38%
Age	59.6 (mean)	Age range 18–88 years
Caucasian	58/98	59%
Black	25/98	25%
Other (Race)	15/98	15%
Diabetes mellitus	54	63%
Predialysis	28/98	29%

**Fig. 2:**
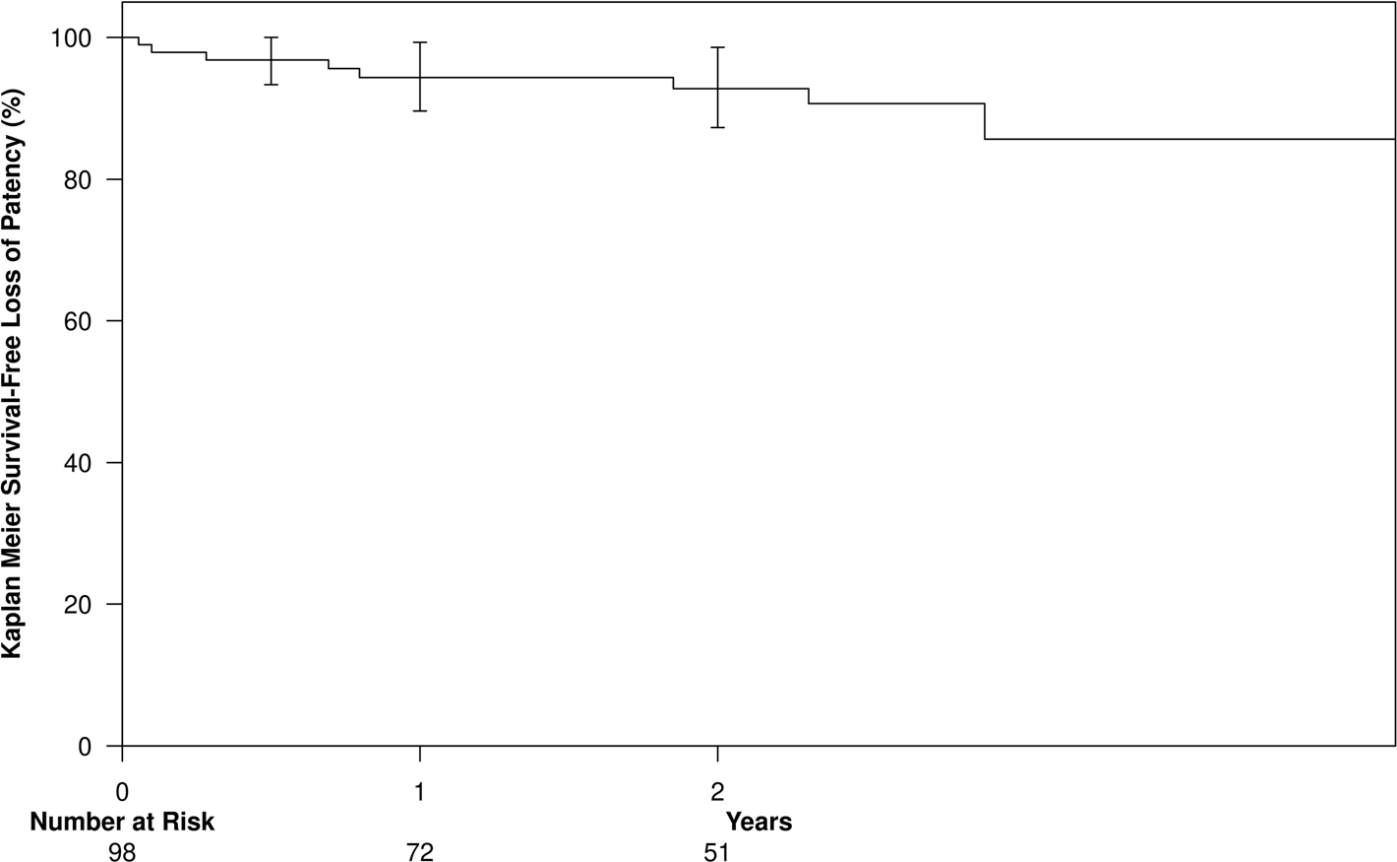
Cumulative patency

**Fig. 3:**
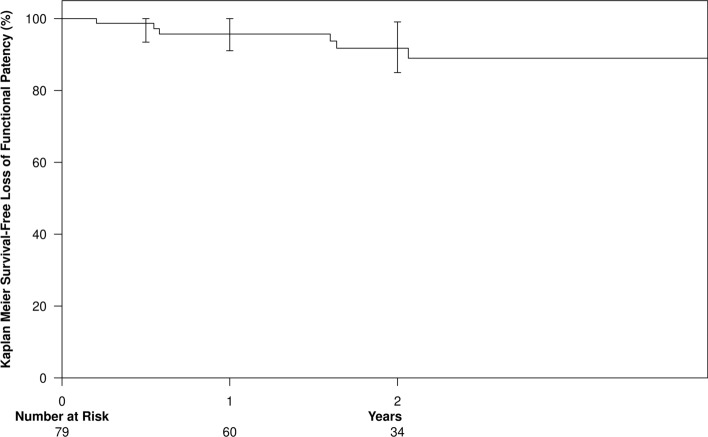
Functional patency

Two-year cumulative patency was 94.3% at 12 months and 92.7% at 24 months (95% confidence interval: 77.1–99.8) (Fig. 2). Two-year functional patency was 95.7% at 12 months and 91.7% at 24 months with a mean follow-up time of 803 days (Fig. 3). None of the variables which were examined in the study (e.g., fistula location, brachial artery blood flow before and after fistula creation, or patient demographics) showed any significant impact on the two-year cumulative or functional patency curves (Tables 2 and 3). Median time to maturation was 95 days (Interquartile range 51–231 days) with male gender and brachial vein coiling at index procedure associated with higher probability of maturation (p = 0.01 and 0.02, respectively) (Table 4). There were 34 censored events with four patients undergoing renal transplantation and 30 patients deceased unrelated to the endoAVF (sepsis unrelated to the endoAVF, cardiac arrest, multisystem organ failure, or respiratory failure).

**Table 2:** Univariate analysis of patient and endoAVF variables on cumulative patency

Variate		Hazard ratio (95% CI)	*P* value
Laterality	Left	1.96 (0.4–9.8)	0.41
	Right	1.0	
Sex	Male	0.93 (0.22–3.90)	0.92
	Female	1.0	
Diabetes mellitus	Yes	0.91 (0.15–5.42)	0.91
	No	1.0	
Predialysis	Yes	1.41 (0.33–5.89)	0.65
	No	1.0	
Fistula location	Ulnar artery and vein	2.40 (0.29–19.98)	0.42
	Radial artery and vein	1.0	
Coil embolization during procedure	Yes	1.83 (0.23–15.07)	0.56
	No	1.0	
Brachial artery flow pre-creation	> 60 ml/min (median)	1.96 (0.18–21.62)	0.58
Brachial artery flow post-creation	> 1030 ml/min (median)	0.222 (0.03–1.99)	0.18

**Table 3:** Univariate analysis of patient and endoAVF variables on functional patency

Variate		Hazard ratio (95% CI)	*P* value
Laterality	Left	1.2 (0.22–6.58)	0.83
	Right	1.0	
Sex	Male	0.91 (0.17–5.00)	0.92
	Female	1.0	
Diabetes mellitus	Yes	1.2 (0.11–13.0)	0.89
	No	1.0	
Predialysis	Yes	1.2 (0.22–6.56)	0.83
	No	1.0	
Fistula location	Ulnar artery and vein	3.28 (0.14–79.0)	0.46
	Radial artery and vein	1.0	
Coil embolization during procedure	Yes	1.03 (0.12–8.90)	0.98
	No	1.0	
Brachial artery flow pre-creation	> 60 ml/min (median)	0.84 (0.68–1.34)	0.79
Brachial artery flow post-creation	> 1030 ml/min (median)	0.38 (0.03–4.21)	0.431

**Table 4:** Univariate analysis of patient and endoAVF variables on maturation

Variate		Hazard ratio (95% CI)	*P* value
Laterality	Left	1.01 (0.64–1.58)	0.98
	Right	1.0	
Sex	Male	1.98 (1.26–3.20)	0.01
	Female	1.0	
Diabetes Mellitus	Yes	1.07 (0.65–1.76)	0.78
	No	1.0	
Predialysis	Yes	0.96 (0.59–1.58)	0.87
	No	1.0	
Fistula location	Ulnar artery and vein	1.33 (0.80–2.21)	0.277
	Radial artery and vein	0.75 (0.45- 1.26)	0.277
Coil embolization during procedure	Yes	2.11 (1.15–3.88)	0.02
	No	1.0	
Brachial artery flow pre-creation	> 60 ml/min (median)	1.33 (0.77–2.29)	0.31
Brachial artery flow post-creation	> 1030 ml/min (median)	1.19 (0.73–1.95)	0.49

A total of 144 subsequent interventions were performed for fistula maturation and maintaining functional patency which represented a rate of 0.73 interventions per patient year. Maturation procedures were 12 surgical vein elevations and/or transposition (12.2%), 30 coil embolization procedures (30.6%) remote to the endoAVF creation procedure to enhance flow to the superficial veins, and 2 percutaneous medial vein ligations which were performed quicker than and without the need for venous access for embolization (2%). Maintenance interventions were 86 angioplasties, 9 thrombectomies, and 5 thrombectomies with angioplasty. Twenty-four patients required more than one intervention within two years (range 2–7). One (0.9%) major or Grade 3 complication occurred with pseudoaneurysm formation at the wrist ulnar artery access site from patient noncompliance (failure to follow hemostasis protocol and left hospital against medical advice) requiring later surgical repair. No other complications including infections occurred at the time of procedure or during follow-up.

## Discussion

Given the introduction of the 4Fr WavelinQ system in 2018 that followed the original 6 Fr system, there has been a limited window of time to assess longer-term outcomes for patency and functionality. Furthermore, from the original prospective clinical studies examining outcomes of the 4Fr device, the protocol did not require longer-term follow-up [6]. This retrospective multi-center study with a larger patient population addresses the lack of longer-term outcomes. Furthermore, the patient population presented in this study was not part of any prior clinical trial and reflect predominantly nonacademic practice.

From the two studies published by Berland, the EASE study (Endovascular Access System Enhancements Study) which combined patients from three prospective studies that utilized the 4Fr system had a technical success rate of 100% with an 87% cumulative patency at 6 months [7]. A later post-market approval study (which included the EASE study population) had a technical success rate of 96% with 88% cumulative patency at six months [6]. Patency beyond 6 months was not examined. In another comparative retrospective single-operator study of the Ellipsys and WavelinQ devices by Shahverdyan et al., cumulative patency was not assessed [12]. A single-center retrospective study of 30 patients who had fistulas created with the WavelinQ 4 Fr device had a 100% technical success rate, with an 87% maturation rate with longer-term 96% one-year and 82% two-year cumulative patency rates [8]. Cumulative patency at 2 years was 93% in this study.

Functional patency was 93% at two years indicating that once two-needle cannulation was achieved it was maintained with a high level of longer-term durability, which was not ascertained in the prior published studies. Interventions per patient year were 0.73 that included maturation interventions. While the intervention rate is similar to Kitrou’s 0.53 and the post-market approval study of 0.55 [6, 8], the relatively low rate of interventions highlights the need for minimal intervention to maintain functional patency up till two years. By comparison, surgically created accesses require approximately 1.9 interventions per patient year [2]. Time to maturation ranges from 41 to 130 days in prior studies [6, 8, 12, 13], whereas we observed median maturation time of 95 days. Although within the observed range, the later time to maturation may be the result of an inconsistent follow-up protocol between the different sites and the COVID pandemic impacting the ability to evaluate patients in person.

Univariate analysis of collected variables did not identify any factors that influenced cumulative or functional patency. This included an assessment of ulnar artery/ulnar vein and radial artery/radial vein fistulas indicating that fistula location did not impact patency. However, improved maturation was associated with male gender and coil embolization during endoAVF creation. Improved maturation in male patients has also been observed with surgical AVF creation [14, 15]. Twenty-five percent of patients (25/98) in this study were black, a larger percentage than prior study populations with the WavelinQ device yet ethnicity did not influence patency or maturity.

The selection of the vein for embolization was guided by flow rate observations during the verification of fistula creation. Coil embolization was performed in 80.4% of patients to enhance blood flow through the new AVF, a number that is higher than presented in Kitrou’s study, where coil embolization was performed in 33.3% of the cases [8]. This observation is likely related to operator preference as the decision to embolize is somewhat subjective based on observed flow patterns post-endoAVF creation and may be related to site of fistula creation. Major complications were rare, with only one instance observed—pseudoaneurysm formation at the wrist ulnar artery access site. This was the likely result of patient noncompliance during post-procedure recovery. No infectious complications were observed, whereas a 4.1% rate was observed with surgical fistulas in a meta-analysis [16]. The low complication rate correlates with previous studies with 1–4 complications reported overall [6, 8, 17].

Regarding technical challenges observed during this study period, patients with calcified arteries at the intended site of endoAVF creation should be avoided. It is believed that the technical failures in this study were attributed to arterial calcification, and it is unclear what degree of calcification presents a problem. Also, the authors gravitated to brachial nerve blocks as endoAVF experience matured. Brachial blocks reduce vascular spasm, lead to vessel dilation, and likely reduce the IV analgesia requirements. Patience is also required for retrograde venous access as traversing venous valves can be difficult. For patient safety, arterial access obtained at wrist level resulted in no arterial access complications with the exception of one event described above.

A direct comparison to the Ellipsys endoAVF device (Medtronic, Minneapolis, MN) may not be appropriate given differences in the location of fistula creation and energy used to create the fistula (between the radial artery and the venous perforator; thermal energy) and that longer-term outcomes are from patients selected prospectively for the pivotal US prospective study initiated in 2015. However, in 85 patients cumulative and functional patency at 2 years was 88% and 92%, respectively, which are similar to outcomes observed in this study suggesting that endoAVF creation is a durable access solution [18]. Intervention rates are not comparable as the Ellipsys study intervention rate did not include interventions within the first two years of creation for those patients who had a mature fistula. Also, a comparison to surgically created autogenous fistulas is limited by differing definitions, patient populations, and fundamental differences in the anatomic locations of endoAVFs. Despite these considerations, a meta-analysis of surgically created AVFs found a primary failure rate of 23%, with subsequent patency rates of 71% and 64% at the one- and two-year marks, respectively [19]. Another surgical fistula meta-analysis found one-year cumulative patency to be 79% [16]. In this study, if the cases of failure to mature (intent to treat) are included, one- and two-year cumulative patency would be 84% and 82%.

This study has several limitations. Given the retrospective nature of the study and nonconformity in data points collected pre- and post-procedure, certain variables could not be collected that may be relevant to outcomes such as the patients prior access history, time to dialysis catheter removal, vein diameters pre fistula creation, and brachial artery flow volumes over time. Also, all three centers had different follow-up algorithms which likely influenced time to maturation and functional patency.

## Conclusion

The utilization of the WavelinQ system for endoAVF creation demonstrated positive outcomes for technical success and a two-year cumulative and functional patency. The need for minimal interventions to maintain patency further supports the utility of endoAVFs as a resilient and dependable access solution for hemodialysis patients and should be considered before above elbow access options.
